# Genome-Wide Discovery and Information Resource Development of DNA Polymorphisms in Cassava

**DOI:** 10.1371/journal.pone.0074056

**Published:** 2013-09-11

**Authors:** Tetsuya Sakurai, Keiichi Mochida, Takuhiro Yoshida, Kenji Akiyama, Manabu Ishitani, Motoaki Seki, Kazuo Shinozaki

**Affiliations:** 1 RIKEN Center for Sustainable Resource Science, Tsurumi-ku, Yokohama, Kanagawa, Japan; 2 RIKEN Biomass Engineering Program, Tsurumi-ku, Yokohama, Kanagawa, Japan; 3 Kihara Institute for Biological Research, Yokohama City University, Totsuka-ku, Yokohama, Kanagawa, Japan; 4 Agrobiodiversity Research Area, International Center for Tropical Agriculture (CIAT), Cali, Colombia; Leuven University, Belgium

## Abstract

Cassava (*Manihot esculenta* Crantz) is an important crop that provides food security and income generation in many tropical countries, and is known for its adaptability to various environmental conditions. Its draft genome sequence and many expressed sequence tags are now publicly available, allowing the development of cassava polymorphism information. Here, we describe the genome-wide discovery of cassava DNA polymorphisms. Using the alignment of predicted transcribed sequences from the cassava draft genome sequence and ESTs from GenBank, we discovered 10,546 single-nucleotide polymorphisms and 647 insertions and deletions. To facilitate molecular marker development for cassava, we designed 9,316 PCR primer pairs to amplify the genomic region around each DNA polymorphism. Of the discovered SNPs, 62.7% occurred in protein-coding regions. Disease-resistance genes were found to have a significantly higher ratio of nonsynonymous-to-synonymous substitutions. We identified 24 read-through (changes of a stop codon to a coding codon) and 38 premature stop (changes of a coding codon to a stop codon) single-nucleotide polymorphisms, and found that the 5 gene ontology terms in biological process were significantly different in genes with read-through single-nucleotide polymorphisms compared with all cassava genes. All data on the discovered DNA polymorphisms were organized into the Cassava Online Archive database, which is available at http://cassava.psc.riken.jp/.

## Introduction

Cassava, *Manihot esculenta* Crantz (2*n = *36), is a tropical crop that is important for food security in tropical regions worldwide [1]. The Food and Agriculture Organization of the United Nations (FAO) reports that over 200 million tons of cassava is produced per year, and cassava serves as the primary food source for millions of people. The starch extracted from cassava root is used as a raw material for a wide range of food products and industrial goods, including paper, cardboard, textile, plywood, glue, and alcohol [2]. Moreover, because starch production from cassava is inexpensive compared to that from other crops, it is gaining attention as a biomass source for fuel production [3]. By virtue of its remarkable tolerance to abiotic stresses, cassava is grown in marginal, low-fertility acidic soils [4]. It is known to maintain a healthy appearance in drought-prone areas, remaining photosynthetically active, albeit at a reduced rate [5]. Because cassava is highly drought resistant and the tubers can be retained in the soil for a couple of years, it is considered an important reserve carbohydrate source for the prevention of or relief from famine [6].

Accumulation of nucleotide sequence information from various organisms, including cassava, has been promoted as an effective method for gene discovery in recent decades [7]. The development of several full-length cDNA and expressed sequence tag (EST) collections has led to functional genomics studies in several plant species [8]–[13]; moreover, full-length cDNAs have been utilized to develop comprehensive transgenic lines of Arabidopsis and rice [14]–[16]. Large-scale cassava cDNA collection projects have been conducted by various cassava research groups [17]–[20], information resources from which have been used in transcriptomics research [21]–[23]. The cassava draft genome sequence is now publicly available, and the initial assembly spans 419.5 Mb, covering 54% of the estimated cassava genome size (770 Mb). At present, 30,666 protein-coding loci have been predicted from this genome sequence and 3,485 alternative splice forms are supported by ESTs [24].

Molecular markers are important for plant research and breeding, and are being applied to accelerate effective plant selection through marker-assisted selection, based on genome-level selection of chromosomal segments. In plant genetic research, molecular markers are also being used for the analysis of population structure, the study of evolutionary relationships, and, in sequenced model systems such as Arabidopsis, for studies on the genetic structure of individuals at the whole-genome level [25]. In addition, single-nucleotide polymorphism (SNP) markers have recently gained interest in the scientific and plant-breeding communities [26]. SNPs occur as single-nucleotide differences between individuals, and thousands of SNP markers are widely used in animal and human genome analysis [27], suggesting that their more widespread use in plants should be promoted.

Studies on genetic mapping and molecular marker development in cassava have been published [28]–[30], and several studies have focused on the analysis or discovery of simple sequence repeat loci [31], [32] and mapping of quantitative trait loci [33]. To further promote progress in genetics and breeding, higher-density markers, such as SNP markers, are required. SNPs and insertions and deletions (InDels) are common natural mutations in populations [34], [35]. The SNPs and InDels discovered in cassava [36], [37] are quite important for cassava breeding research; cassava is an outcrossing species and produces botanical seed in many environments, but is mainly propagated using stem cuttings. Thus, most cassava cultivars are considered heterozygous, which makes it more difficult to develop molecular markers [24], [38]. Therefore, it is necessary to detect additional DNA polymorphisms using the available cassava genome and transcribed sequences to improve molecular marker development in cassava. DNA polymorphism discovery is important not only for molecular breeding but also for understanding gene function, and elucidation of the relationship between polymorphisms, gene function, and gene duplication should shed light on gene function and evolution [39]–[41].

Many public databases of major plant genomics resources have been constructed to integrate knowledge and to facilitate further research [42]. PlantGDB, TIGR Plant Transcript Assemblies, and HarvEST provide clustered and representative transcript sequences resulting from advances in large-scale EST compilation. They are useful not only for the provision of comprehensive transcripts but also for comparisons among plant species [43]–[45]. The integration of genetic markers with corresponding genomic and/or transcriptomic sequences is already facilitating genome-wide genetic approaches. The Arabidopsis Information Resource (TAIR) is a popular site in the Arabidopsis community [46], and Gramene is a database for monocot plant comparative genomics that provides genetic maps of various plant species [47]. Accordingly, to facilitate cassava research and to assemble relevant knowledge on this crop, we are gathering polymorphism information and relational annotations of the cassava genome into a new database.

Here, we describe the discovery of over 10,000 SNPs and InDels in cassava, and the relationship between polymorphism and gene function. We have organized the results of our research, including gene models and functional annotations, into a database named “Cassava Online Archive,” which can be accessed at http://cassava.psc.riken.jp/.

## Materials and Methods

### Cassava Sequence Dataset

We retrieved cassava sequences from the GenBank EST section in December 2012 [48]. The sequences were first checked for sequence contamination and simple repeats by using the SeqClean script (http://compbio.dfci.harvard.edu/tgi/software/) with the default runtime options. Vector sequences included in these ESTs were then trimmed using the cross_match utility of the Phred/Phrap package with -minmatch 10 -minscore 20 runtime options [49] and the UniVec_Core database of NCBI (http://www.ncbi.nlm.nih.gov/VecScreen/UniVec.html). Contamination was detected via BLASTN sequence similarity searches with the default runtime settings against both the *Escherichia coli* K12 genome (GenBank accession number U00096) and the bacteriophage phi_X174 (GenBank accession number J02482) genome sequences. Sequences with threshold E-values of less than 1e-100 were removed. The cleaned sequences were then classified by cultivar description in each GenBank format file. A portion of the sequences was confirmed by contacting the contact person or submitter of the sequence record (Table 1). To discover polymorphisms and obtain genomic information, we also downloaded the cassava draft genome sequence and the predicted protein and transcript sequences (variety AM560-2, JGI annotation v4.1) from the Phytozome website (http://www.phytozome.net/) [50].

**Table 1:** Sequence summary of DNA polymorphism discovery.

Variety	Number of downloadedsequences	Number of cleanedsequences	Number of assembledsequences
AM560-2^a^	34,151	34,151	22,589
CAS36.01	254	254	249
CAS36.04	488	488	488
CM21772	95	95	89
CM523-7	3,608	3,581	3,495
MCol22	4,764	4,764	4,604
IAC 12.829	63	63	63
KU50 (MTAI16)	35,572	35,500	32,984
MBra685	2,506	2,506	2,355
MCol1522	1,979	1,975	1,854
MNga2	40	40	33
MPer183	3,391	3,388	3,206
Mirassol	210	210	208
SG107-35	720	720	651
Sauti, Gomani, Mbundumali, TME 1 and Mkondezi	5,046	5,046	4,607
TMS30572 and CM2177-2	7	7	5
Unknown	21,888	21,886	19,405
**Total**	**114,782**	**114,674**	**96,885**

a Annotated transcript sequences from the cassava draft genome sequence (JGI annotation v4.1).

### DNA Polymorphism Discovery and Primer Design

The sequences obtained by the above process and the predicted transcript sequences from the cassava draft genome sequence were assembled using the CAP3 program with the default runtime options [51]. Polymorphisms (SNPs and InDels) were discovered from the contig sequence alignment according to the following criteria: (i) The contig could be aligned with the cassava draft genome sequence [24]; (ii) The nucleotide at the polymorphism site was not N; (iii) The SNP consisted of 2 types of nucleotides (to avoid false SNP detection due to cross-contamination with other loci in the contig sequence alignment); (iv) The polymorphism was supported by at least 2 sequences in a cassava variety; (v) The nucleotide at the polymorphism site was the same in the contig sequence alignment of each variety; (vi) There were fewer than 3 other discontinuous nucleotide polymorphisms around 5 bp of a SNP site (to prevent false SNP detection by low-quality sequences).

The physical position of each SNP or InDel was deduced from the cassava draft genome sequence. Primer pairs were then designed to amplify the genomic region around each discovered SNP or InDel site using Primer3 (Release 2.2.3) [52] with the following conditions: primer size, 18–25 bp; product size, 150–200 bp; GC content, 45%–65%; and melting temperature, 58–72°C. The input file of R_MesP_000003m.00 is provided in Text S1 as an example.

### Validation of Discovered SNPs

To validate the SNPs discovered in this study, we compared them with the validated SNP information published in a previous report [37]. We first downloaded the online resources, including the validated SNP information, from the journal website. We then extracted the SNP validation result and the information on the SNP locations and alleles in each contig sequence from the online resources. Next, we set up the physical locations of the validated SNP sites on the draft genome sequence by combining the SNP locations in each contig sequence with the gene annotation information from the draft genome sequence. Finally, we validated the locations and alleles of the SNPs discovered in this study by comparing them with the information on the previously validated SNPs.

### DNA Polymorphism Characterization

Transition-to-transversion ratios, nonsynonymous and synonymous substitutions, and premature stop and read-through SNPs were examined using custom Perl scripts that analyzed the sequence assembly and the annotated transcript sequences. SNPs located within cassava gene models (JGI annotation v4.1) were classified based on the positions of the 5′-untranslated region (UTR), 3′-UTR, and protein-coding sequence (CDS) within the gene model. SNPs detected in cassava predicted transcript sequences were examined for the relevant codon as well as for nonsynonymous and synonymous substitutions. SNPs were further annotated according to whether they produced a premature stop codon or disabled a stop codon. For the assignment of a cassava gene model to a Pfam protein domain [53], we used a part of JGI annotation v4.1 (Mesculenta_147_annotation_info.txt).

For gene ontology (GO) [54] term assignment, all cassava gene models were aligned to Arabidopsis gene models [46] by using BLASTP [55]. To support the assignment correctness between cassava and Arabidopsis genes, the best alignment hits with over 70% alignment coverage from Arabidopsis gene model to cassava gene model (alignment length/Arabidopsis protein length in a BLASTP pairwise alignment) and E-values less than 1e-5 were used for correspondence between cassava genes and Arabidopsis genes. By using Arabidopsis locus IDs relevant to each cassava gene, cassava genes were assigned to TAIR GO Slim. Next, to confirm the proportion independency of GO assignment between two groups (premature stop SNP gene versus all genes, and read-through SNP gene versus all genes), we performed a Pearson chi-square test on each group set, followed by residual analysis of each GO term to identify GO terms with significant difference.

### Statistics Analysis

To confirm the independence of GO assignments between the two groups, we used the Pearson chi-square test with the adjusted standardized residual method [56]. This statistical method was used to compare frequencies and to determine an indication of the strength of independence for each shared GO term between the two groups. The chi-square test indicates whether independence exists between two grouped variables, but it does not indicate the strength of the association per variable. It is necessary to identify the GO terms having larger differences between the observed and expected frequencies. These differences are referred to as residuals, and can be standardized and adjusted to follow a normal distribution with mean 0 and standard deviation 1 [57]. The expected frequencies (E_ij_) and adjusted standardized residuals (d_ij_) are given by the following equations:
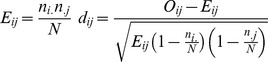
where, O_ij_ is the observed frequency in the cell in row i (e.g. GO functional category) and column j (e.g. group of read-through SNP genes or all genes); n_i.,_ total frequency for row i (e.g. total of observed frequencies for a GO functional category); n_.j_, the total frequency for column j (e.g. total of observed frequencies for a group such as the group of read-through SNP genes); N is the overall total frequency; and E_ij_ is the expected frequency in the cell in row i and column j. The larger the absolute value of the adjusted standardized residual, the larger the difference between the observed and expected frequencies. An absolute value of the adjusted standardized residual of >1.96 indicates that a GO term is significantly different between the grouped variables with a significance level of 0.05. Similarly, an absolute value of >2.575 indicates that a GO term is significantly different between the grouped variables with a significance level of 0.01.

## Results and Discussion

### Dataset and DNA Polymorphism Discovery

We retrieved 80,631 cassava sequences from GenBank [48] and cleaned them prior to classifying them by cassava variety. We obtained a total of 80,523 sequences from 16 cassava varieties or libraries (CAS36.01, CAS36.04, CM21772, CM523-7, MCol22, IAC 12.829, KU50/MTAI16, MBra685, MCol1522, MNga2, MPer183, Mirassol, SG107-35, ‘Sauti, Gomani, Mbundumali, TME 1, and Mkondezi’, ‘TMS30572 and CM2177-2’, and variety unknown). The distribution of the cleaned sequences is shown in Figure S1. We appended the predicted transcript sequences from the cassava draft genome sequence (variety AM560-2) to the classified sequence set, and obtained a total of 114,674 sequences as the starting data for this study (Table 1).

The sequences were assembled using the CAP3 [51] program, which produced 16,363 contigs and 17,789 singlets. We used the alignment of the 96,885 cleaned sequences, which formed the contigs (Table 1). The numbers of sequences per assembled contig were between 2 and 707, with an average of 5.9 sequences per contig (Figure S2). An overview of polymorphism detection and the average number of polymorphisms per contig among contigs in which polymorphisms were detected is shown in Figure S3. The average number of polymorphisms per contig was 3.8, and the average per fraction ranged from 2.6 to 6.2 polymorphisms/contig. This analysis suggests that the polymorphism detection was unbiased (Figure S3). Analysis of the sequence alignment of the assembled contigs revealed that 10,546 SNPs and 674 InDels were discovered using a custom script (see Materials and Methods). No polymorphisms were identified in the cassava variety sets MNga2 and ‘TMS30572 and CM2177-2’, because of the small numbers of sequences in these sets. With regard to InDel length, the numbers of 1-, 2- and 3-nucleotide InDels were 612, 42, and 20, respectively (Figure S4).

For 8,794 of the 10,546 SNPs discovered, we succeeded in designing a PCR primer pair for amplification of the genomic region. SNPs were discovered in 3,252 genes, with an average of 3.2 SNPs per gene and a SNP frequency of 1 SNP per 1,072.5 bp in transcripts, including introns (Table 2). Similarly, for 522 of the 674 InDels identified, we designed PCR primer pairs to amplify the genomic region. InDels were identified in 583 genes, with an average of 1.2 InDels per gene and an InDel frequency of 1 InDel per 3,291.4 bp in transcripts, including introns (Table 2). Based on the number of polymorphisms discovered in this study and the genes predicted from the cassava genome sequence, approximately 100,000 SNPs and 40,000 InDels are found in the transcribed regions of the cassava genome.

**Table 2:** Overview of discovered SNPs and InDels.

Type of Polymorphism	SNPs	InDels	Total
Number of polymorphisms	10,546	674	11,220
Number of polymorphisms with designed primer pairs	8,794	522	9,316
Number of genes with polymorphisms	3,252	583	3,402
Average polymorphisms per gene	3.2	1.2	3.3
Average polymorphism interval in transcribed sequences (bp)	337.7	1,012.0	378.2
Average polymorphism interval in genic regions (including introns) (bp)	1,072.5	3,291.4	1,205.8

SNP, single-nucleotide polymorphism; InDel, insertion and deletion.

SNPs have been previously identified in cassava [36], [37]. In this study, we improved the detection of DNA polymorphisms by using a computational approach. This is the largest-scale study of DNA polymorphisms in this crop, and facilitates the understanding of DNA polymorphic tendency as well as of molecular marker development. Moreover, we have organized all the DNA polymorphism information into an internet database named the “Cassava Online Archive,” which is available at http://cassava.psc.riken.jp/.

### Validation of Discovered SNPs

We validated the physical locations on the cassava draft genome sequence and alleles of the SNPs discovered in this study using the information from a previous paper, which reported SNP detection and validation [37]. We set up the information on the locations and alleles of the 1,190 validated SNPs using the online resources described in the previous paper [37]. Of these validated SNPs, 103 were assigned to the locations of the SNPs discovered in the present study. All the alleles were also identical to those in this study (Table S3). Therefore, this result suggests that the SNP detection method of this study is valid.

### SNP Distribution and Polymorphic Genes between Cassava Varieties

Six possible types of single-nucleotide changes were distinguished: C/T, G/A, A/C, A/T, C/G, and T/G, accounting for 2,893, 2,952, 1,108, 1,285, 996, and 1,312 SNPs, respectively. The total numbers of transitions (C/T or G/A) and transversions (C/G, A/T, C/A or T/G) were 5,845 and 4,701, respectively (Table 3). The transition-to-transversion ratio was 1.24, which is comparable to that (1.27) obtained in a previous study [37].

**Table 3:** Summary of the six types of discovered SNPs (transitions and transversions).

Transitions	**C/T**	**2,893**
	G/A	2,952
	**Total**	**5,845**
**Transversions**	A/C	1,108
	A/T	1,285
	C/G	996
	G/T	1,312
	**Total**	**4,701**

Each SNP was classified based on the base change that occurred. The total number of transitions (5,845) is marginally greater than the total number of transversions (4,701), yielding a transition-to-transversion ratio of 1.24.

In 8 cassava varieties (AM560-2, CM523-7, MCol22, KU50, MBra685, MCol1522, MPer183, and SG107-35), from which more than 500 sequences were collected, we conducted further analyses of SNPs for each possible pairwise combination of varieties (Table S1). A polymorphism ratio for all pairwise varieties was calculated using the number of common genes between the 2 varieties relative to all the SNPs found across all varieties (Table 4). This result sheds light on the genetic differences among the cassava varieties, which could provide significant information as a starting point for cassava genetics studies and molecular breeding; this, for example, would contribute to more efficient selection of mapping populations and quantitative trait loci analysis.

**Table 4:** Polymorphism ratio of genes with allelic SNP by pairwise comparison of cassava varieties.

	AM560-2	CM523-7	MCol22	KU50	MBra685	MCol1522	MPer183
**CM523-7**	0.67						
**MCol22**	0.85	0.74					
**KU50**	0.82	0.54	0.66				
**MBra685**	0.40	0.52	0.67	0.58			
**MCol1522**	0.40	0.50	0.65	0.59	0.41		
**MPer183**	0.73	0.61	0.64	0.65	0.61	0.39	
**SG107-35**	0.72	0.50	0.40	0.50	0.50	0.45	0.71

Cassava varieties in which the number of sequences was less than 500 were omitted from this ratio calculation.

### DNA Polymorphism Characterization

As described above, in total, 10,546 SNPs and 674 InDels were discovered (Table 2). We found that 6,613 of the discovered SNPs were located in the predicted CDSs, and SNPs appeared more frequently in CDSs than in UTRs. By contrast, InDels appeared more frequently in UTRs than in CDSs (Table 5). These results are similar to those reported in Arabidopsis [25].

**Table 5:** Annotations of SNPs in the gene models predicted from the draft genome sequence of variety AM560-2.

Annotation	SNP	InDel
CDS (nonsynonymous/synonymous)	6,613 (3,095/3,518)	123
5′-UTR	1,466	188
3′-UTR	2,467	363
**Total**	**10,546**	**674**

CDS, coding sequence; SNP, single-nucleotide polymorphism; InDel, insertion and deletion; UTR, untranslated region.

We next calculated the codon position for each SNP in a CDS. The numbers of SNPs located at the first, second, and third bases of a codon were 1,638, 1,240, and 3,735, respectively (Table S2). More than half the substitutions were located in the third base of a codon, and this result is similar to that of tomato [58]. Given that a change in the third base of a codon has a smaller effect on nonsynonymous amino acid changes than on the other changes, and selective pressure in coding regions reduces the number of nonsynonymous substitutions, it is reasonable that redundancy of the genetic code is mainly observed in the third base of codons.

To clarify how many synonymous changes could potentially affect protein functions in the deduced cassava proteome, we focused on comparative analysis of CDSs regions. SNPs were first classified according to whether they caused no amino acid change (synonymous) or one amino acid change (nonsynonymous). The percentage of nonsynonymous SNPs was 46.8% (Table 5 and Table S2), similar to the results of analyses in Arabidopsis (45.3%) [59], tomato (46.3%) [58], and human (46.5%) [60], but lower than the percentage in rice (56.2%) [61]. In order to examine relationships between nonsynonymous/synonymous changes and protein domains, we then assigned genes with nonsynonymous/synonymous SNPs to gene families based on Pfam protein families [53].

**Figure 1:**
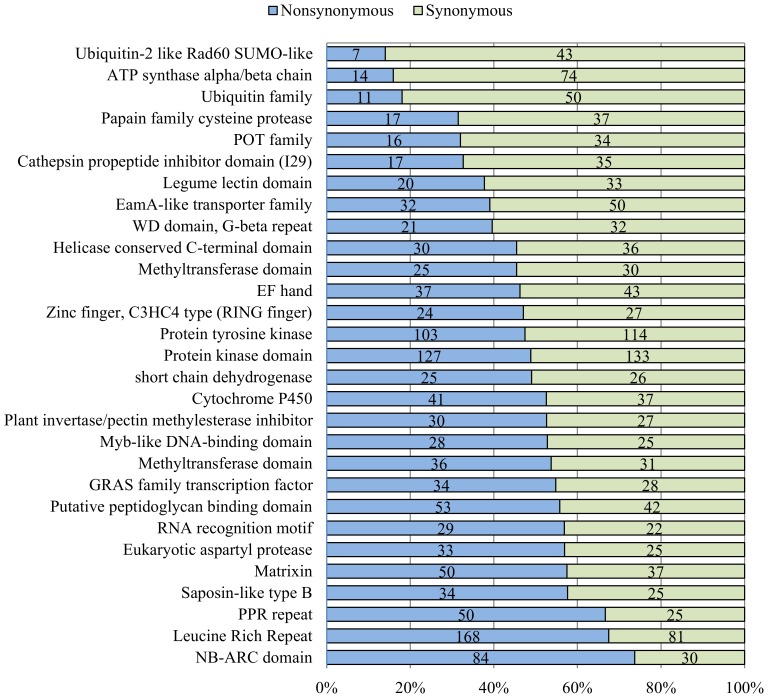
Distribution of nonsynonymous and synonymous single-nucleotide polymorphisms (SNPs). Pfam domains were selected using 30 or more SNPs from nonsynonymous (1,196) and synonymous (1,232) SNPs. Genes encoding members of the ubiquitin family and ATP synthases exhibit lower nonsynonymous-to-synonymous substitution ratios. In contrast, sequences encoding NB-ARC domains and leucine-rich repeats have a significantly higher ratio of nonsynonymous-to-synonymous SNPs.

Genes encoding members of the ubiquitin family and ATP synthases had lower nonsynonymous-to-synonymous substitution ratios. In contrast, sequences encoding NB-ARC domains and leucine-rich repeats had significantly higher ratios of nonsynonymous-to-synonymous SNPs (Figure 1). Domains with higher nonsynonymous-to-synonymous SNP substitution ratios are related to disease-resistance proteins in plants, which is consistent with the diversity of these proteins in response to pathogen infection stress [59], [62]–[65]. Our results suggest that gene families with essential functions (e.g., the ubiquitin family) tend to show substantially lower nonsynonymous-to-synonymous substitution ratios, whereas gene families functioning in regulatory processes and signal recognition, such as the disease-resistance family, have higher nonsynonymous-to-synonymous substitution ratios.

**Figure 2:**
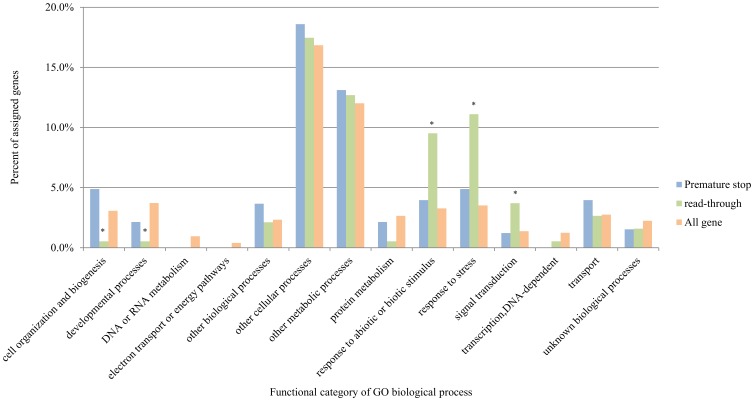
Gene ontology (GO) biological process categories. Gene ontology (GO) biological process categories for all cassava genes containing SNPs that change an amino acid-coding codon to a stop codon (premature stop substitution) and cassava genes containing SNPs that changed a stop codon to a coding codon (read-through substitution). The differences between all cassava genes and read-through SNP genes were supported by p<0.01 according to the Pearson chi-square test. * indicates p<0.05, residual analysis of each GO term.

We identified 24 read-through (change a stop codon to a coding codon) SNPs and 38 premature stop (change a coding amino acid to a stop codon) SNPs (Table S2). By assignment of these genes with SNPs related to ORF structures to GO terms [54], we found that the GO biological process terms “response to abiotic or biotic stimulus”, “response to stress”, “signal transduction”, “cell organization and biogenesis” and “developmental processes” were significantly (p<0.05 on Pearson chi-square test between the 2 groups and p<0.05 residual analysis for each GO term) different in genes with read-through SNP compared to all cassava genes (Figure 2). SNPs in protein-encoding sequences could be important for adaptation to environmental changes, and to obtain a useful trait, an amino acid change in the genome may influence protein structure and function. There was no significant difference upon comparison between genes with premature stop SNPs and all cassava genes (p≥0.05 of Pearson chi-square test).

### Design and Interface of the Cassava Online Archive Database

**Figure 3:**
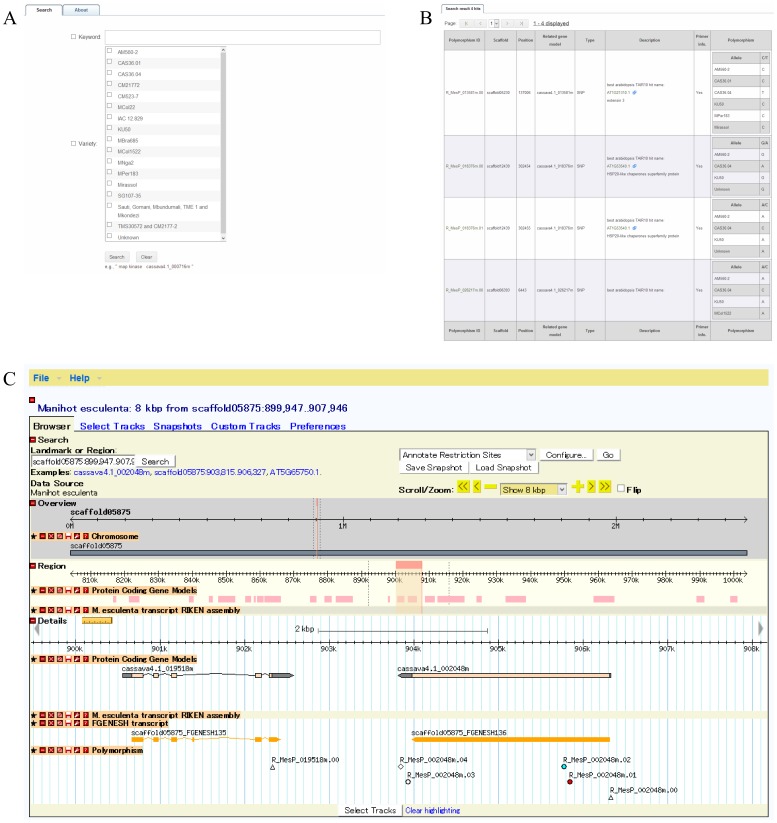
Search interfaces of the Cassava Online Archive database. Users can search for polymorphisms not only using the polymorphism identifier but also using various types of strings, such as a keyword, gene model identifier, and cassava variety name (**A**). The polymorphism records based on search criteria are listed (**B**). The user can also browse target information using the Generic Genome Browser (**C**).

**Figure 4:**
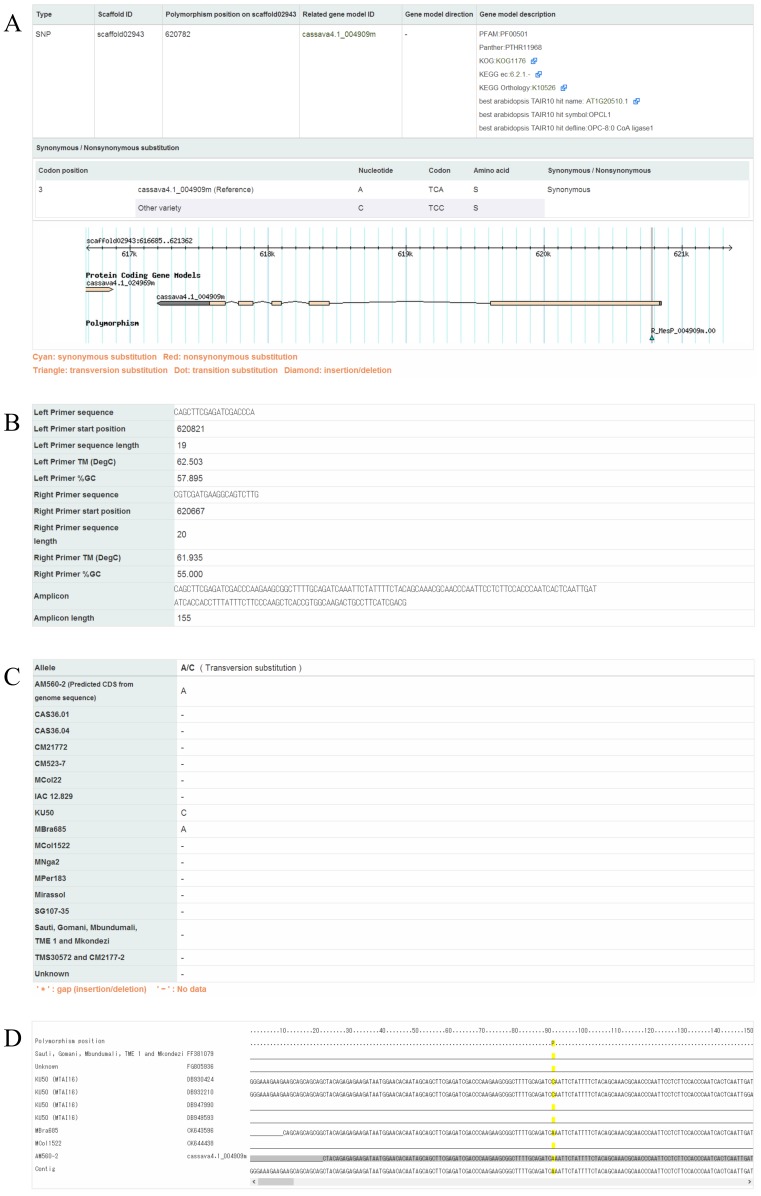
Example of a polymorphism detail. **A.** Genomic information and physical view on the genome browser. **B.** Primer pair information. **C.** Allele per cassava variety. **D.** Sequence alignment for polymorphism detection.

The DNA polymorphism information collected in this study was organized into the Cassava Online Archive database (http://cassava.psc.riken.jp/). To access SNP/InDel records, the Cassava Online Archive provides for web-based searching by keyword, polymorphism identifier, gene model identifier (e.g., Cassava4.1_004909m), and cassava variety name; target information can also be browsed using the Generic Genome Browser [66] (Figure 3). The web interface of the Cassava Online Archive also contains detail pages for each discovered SNP/InDel, including the location (scaffold ID and physical position), related gene model ID, and allele per variety or library. Moreover, the detail page provides information on the designed primer pair (left/right primer sequence, length, melting temperature, GC content, and the expected amplicon sequence and length). Contig alignments used in the polymorphism discovery process are also provided on the detail page in addition to evidence for SNP/InDel detection. Contig alignments were assembled using the CAP3 program, and contain information on the polymorphism site and sequences per cassava variety or library (Figure 4). For the purpose of facilitating cassava studies, the database houses information on the custom oligo-microarray that we previously developed and the gene annotation related to the designed microarray probes [23].

## Conclusions

Our study generated comprehensive SNP and InDel data from cassava ESTs and the draft genome sequence of various varieties of cassava. By mining the resulting polymorphisms in detail, we were able to more accurately understand the classification and annotation of the position of each polymorphism with respect to the coding regions of each gene. Our results shed light on the relationship between nonsynonymous and synonymous substitution genes. Moreover, we designed primer pairs for the amplification of polymorphisms to facilitate molecular marker development. Finally, we organized and integrated our results into a web-based database, meeting the demands of researchers seeking information related to cassava genes. We believe that the Cassava Online Archive database will facilitate cassava genomics research and contribute to molecular breeding.

## Supporting Information

Figure S1 **Distribution of the sequences used for detecting DNA polymorphisms.**
(TIFF)

Figure S2 **Distribution of the contigs and sequences in the assembly for detecting DNA polymorphisms**. The numbers of sequences per assembled contig were between 2 and 707 with an average of 5.9 sequences per contig.
(TIFF)

Figure S3 **Overview of polymorphism detection and average number of polymorphisms per contig in which polymorphisms were detected.** The average number of polymorphisms per contig was 3.8, and the average per fraction ranged from 2.6 to 6.2 polymorphisms/contig.
(TIFF)

Figure S4 **Distribution of the InDel lengths**. The numbers of 1-, 2-, and 3-nucleotide InDels (insertions and deletions) are 612, 42, and 20, respectively.
(TIFF)

Table S1 Summary of genes with allelic SNPs detected by pairwise analysis of cassava varieties
(DOC)

Table S2 Details of SNPs located in predicted CDSs from the cassava draft genome sequence
(XLS)

Table S3 Details of SNPs located in predicted CDSs from the cassava draft genome sequence Of the 1,190 validated SNPs by Ferguson et al. in 2013, 103 were able to be assigned to the locations of the discovered SNP in this study. All the alleles were also identical to the result of this study.
(XLS)

Text S1 **Example of an input file during a Primer3 program run.**
(TXT)
