# Supplementary figures and images for: Genome-Wide Discovery and Information Resource Development of DNA Polymorphisms in Cassava
Source: PLoS One. 2013 Sep 11;8(9):e74056. doi: 10.1371/journal.pone.0074056 (PMC3770675; doi:10.1371/journal.pone.0074056)

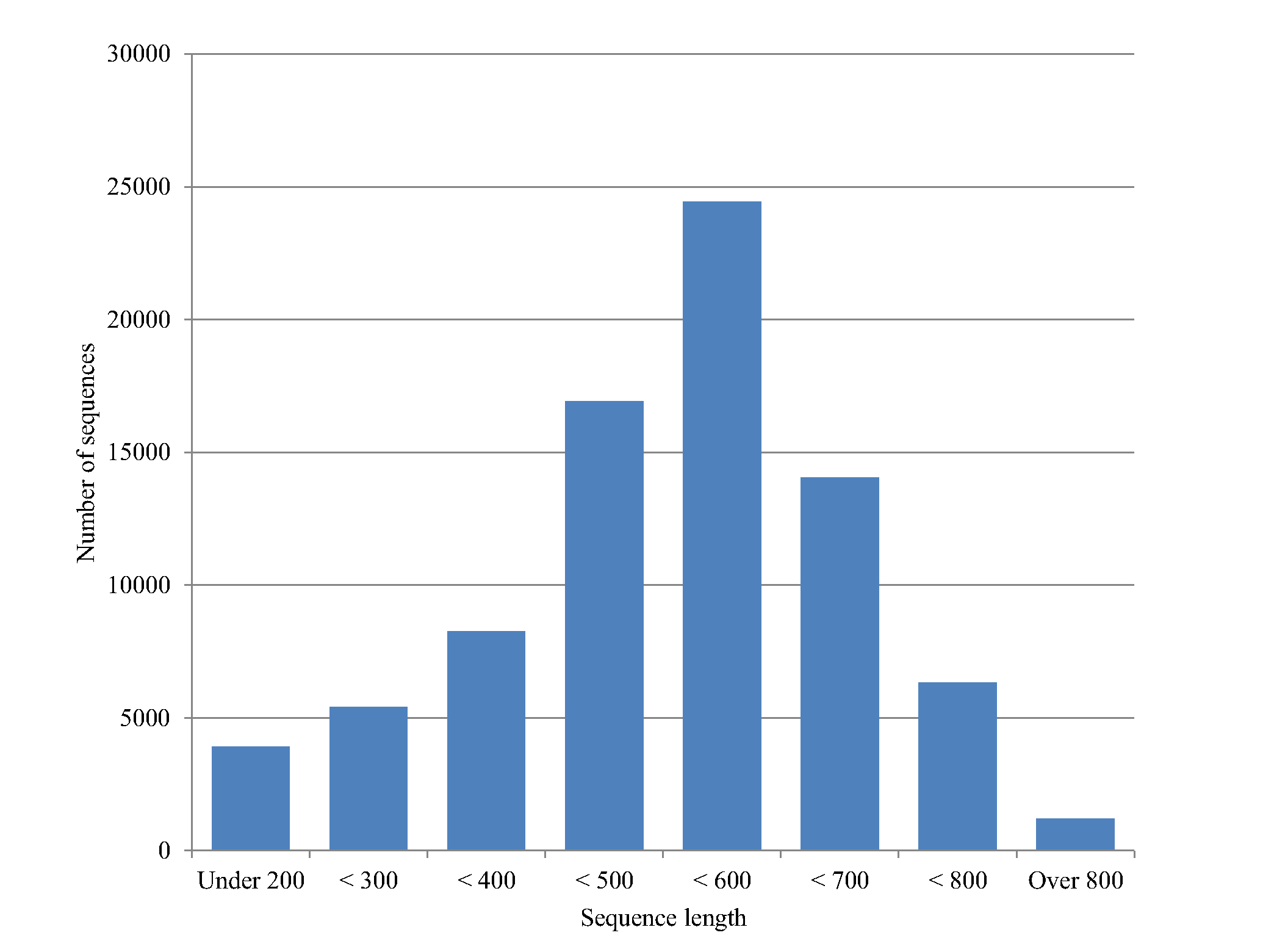

Supplement: Figure S1 — Distribution of the sequences used for detecting DNA polymorphisms. (TIFF) [file pone.0074056.s001.tiff]

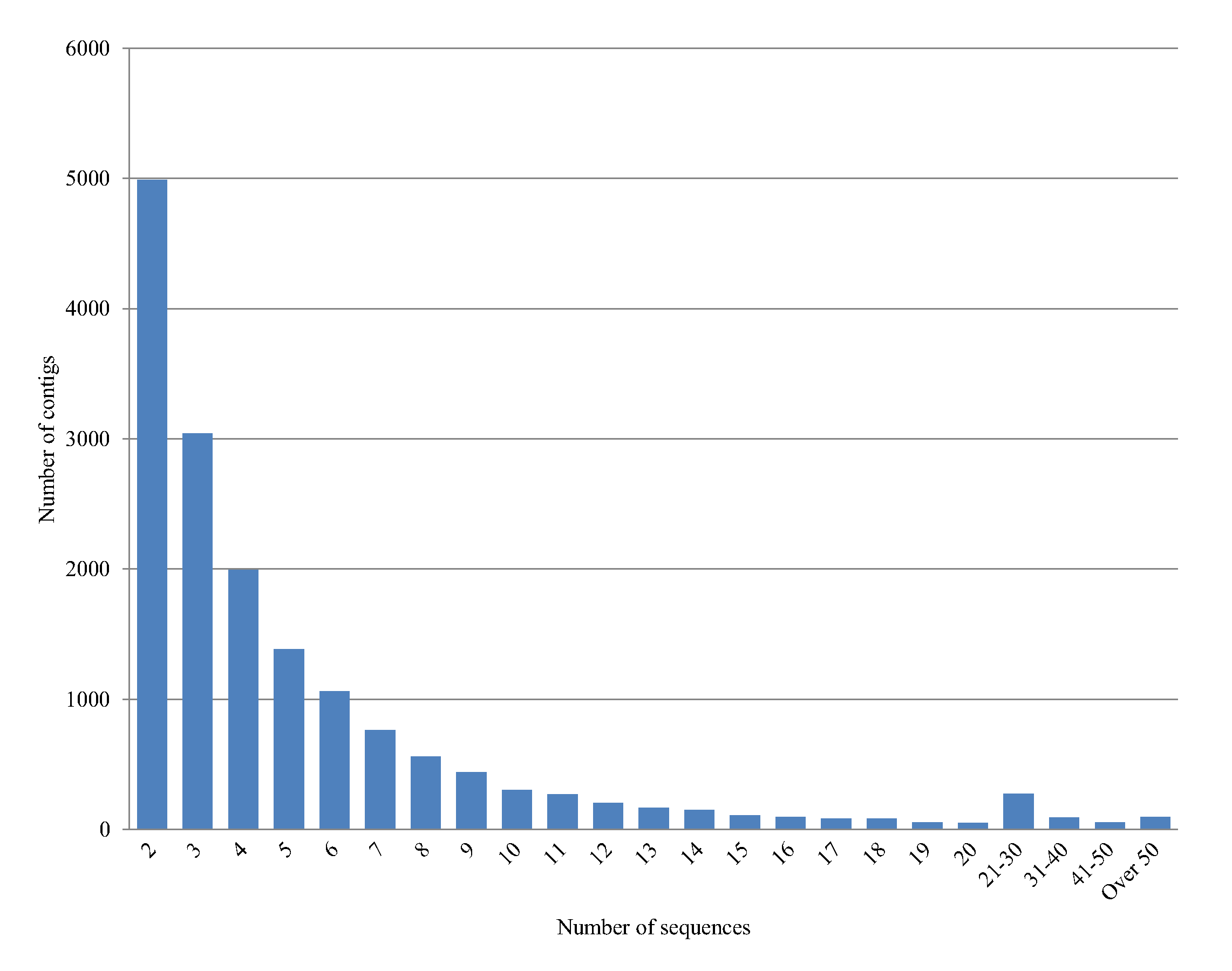

Supplement: Figure S2 — Distribution of the contigs and sequences in the assembly for detecting DNA polymorphisms. The numbers of sequences per assembled contig were between 2 and 707 with an average of 5.9 sequences per contig. (TIFF) [file pone.0074056.s002.tiff]

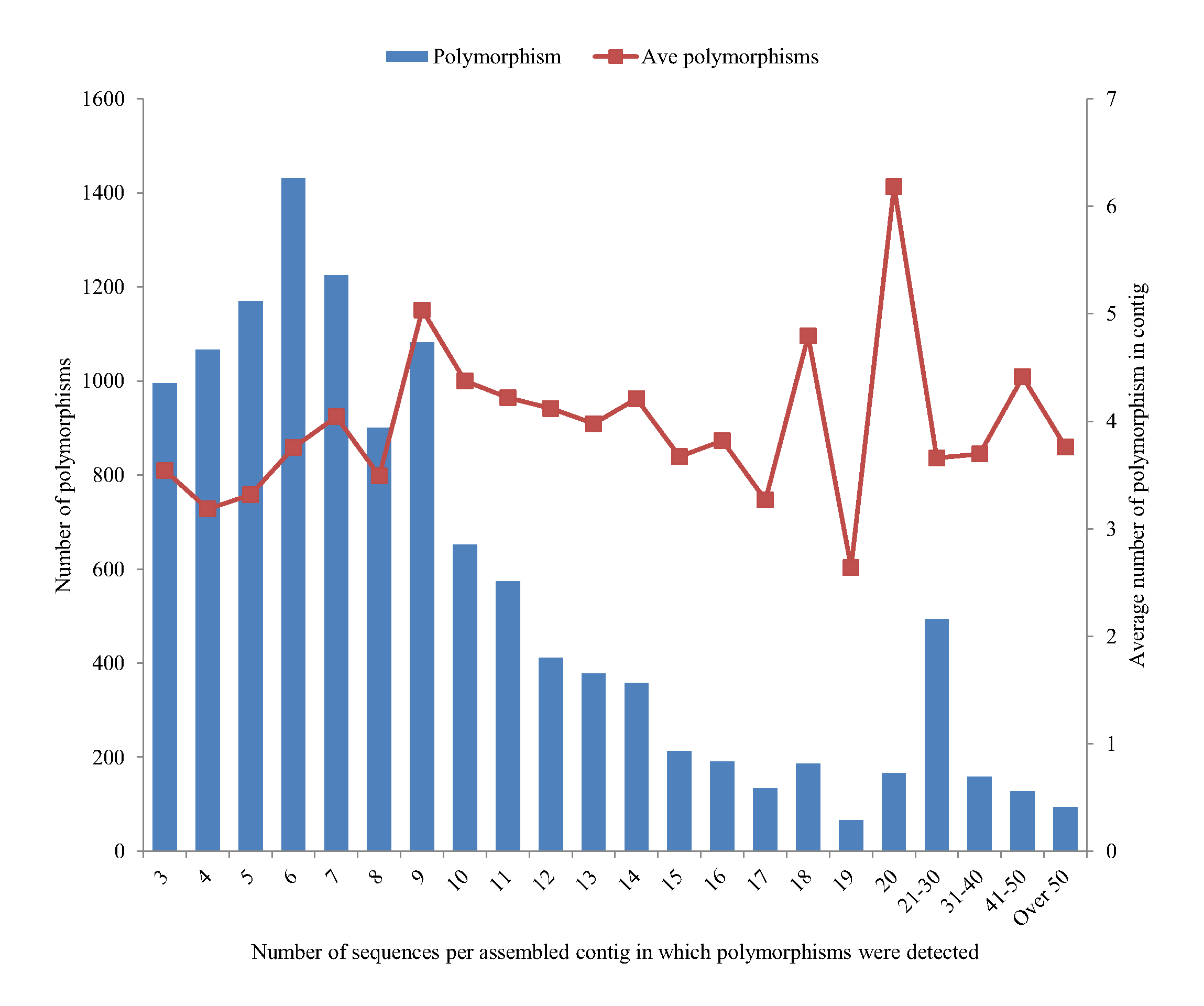

Supplement: Figure S3 — Overview of polymorphism detection and average number of polymorphisms per contig in which polymorphisms were detected. The average number of polymorphisms per contig was 3.8, and the average per fraction ranged from 2.6 to 6.2 polymorphisms/contig. (TIFF) [file pone.0074056.s003.tiff]

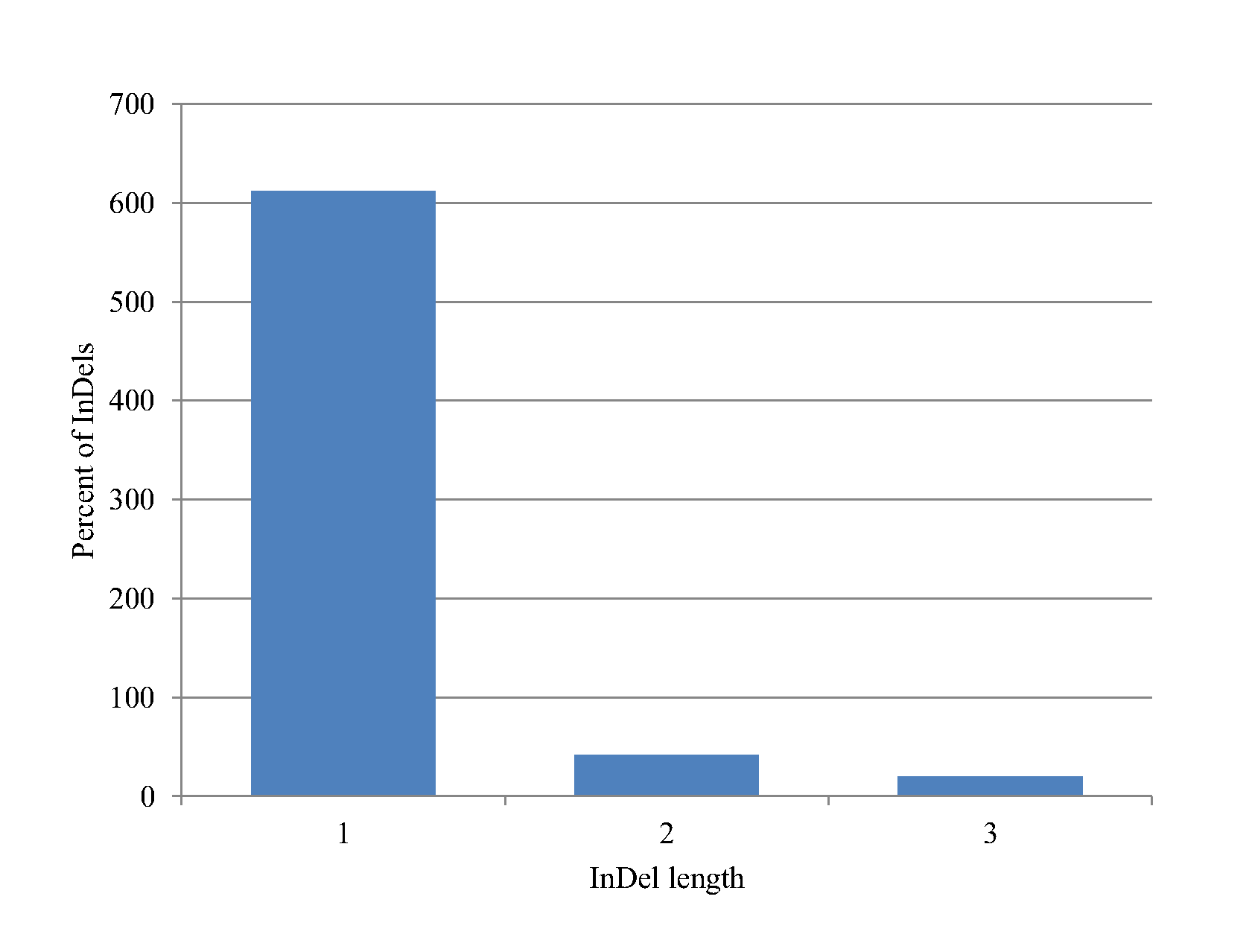

Supplement: Figure S4 — Distribution of the InDel lengths. The numbers of 1-, 2-, and 3-nucleotide InDels (insertions and deletions) are 612, 42, and 20, respectively. (TIFF) [file pone.0074056.s004.tiff]
