# Supplementary material for: Genome-Wide Discovery and Information Resource Development of DNA Polymorphisms in Cassava
Source: PLoS One. 2013 Sep 11;8(9):e74056. doi: 10.1371/journal.pone.0074056 (PMC3770675; doi:10.1371/journal.pone.0074056)
Supplement: Table S1 — Summary of genes with allelic SNPs detected by pairwise analysis of cassava varieties (DOC) [file pone.0074056.s005.doc]

**Table S1.** Summary of genes with allelic SNPs detected by pairwise comparison of cassava varieties

|  | **AM560-2** | **CM523-7** | **MCol22** | **KU50** | **MBra685** | **MCol1522** | **MPer183** |
| --- | --- | --- | --- | --- | --- | --- | --- |
| **CM523-7** | 166/249 |  |  |  |  |  |  |
| **MCol22** | 240/284 | 23/31 |  |  |  |  |  |
| **KU50** | 1792/2182 | 64/118 | 101/154 |  |  |  |  |
| **MBra685** | 75/186 | 15/29 | 20/30 | 61/105 |  |  |  |
| **MCol1522** | 67/166 | 9/18 | 13/20 | 53/90 | 18/44 |  |  |
| **MPer183** | 255/350 | 46/76 | 27/42 | 127/196 | 25/41 | 12/31 |  |
| **SG107-35** | 28/39 | 2/4 | 2/5 | 12/24 | 6/12 | 5/11 | 5/7 |

Each cell indicates the number of genes in which polymorphic single-nucleotide polymorphisms (SNPs) appeared between 2 varieties and the number of genes related to the 2 varieties containing SNPs discovered among all varieties.
